# Coronary Calcium Scanning in Patients after Adjuvant Radiation for Early Breast Cancer and Ductal Carcinoma *In situ*

**DOI:** 10.3389/fonc.2013.00253

**Published:** 2013-09-25

**Authors:** Monique Chang, Jason Suh, Vatsala Kirtani, Andrei Dobrescu, Jonathan Haas, Steven Zeldis, Steven Shayani, Alexander A. Hindenburg

**Affiliations:** ^1^Division of Hematology and Oncology, Winthrop University Hospital, Mineola, NY, USA; ^2^Department of Radiation Oncology, Winthrop University Hospital, Mineola, NY, USA; ^3^SUNY Stony Brook University, Long Island Heart Associates, Mineola, NY, USA; ^4^Mount Sinai Outpatient Services for Long Island, Long Island Heart Associates, Mineola, NY, USA

**Keywords:** calcium scanning, breast cancer, adjuvant radiation, coronary disease, atherosclerosis, agatston, calcium volume score, DCIS

## Abstract

**Background and Objective:** Radiation therapy (RT) is part of standard adjuvant treatment for breast cancer. Earlier studies demonstrated increased cardiac morbidity and mortality from this. Coronary Calcium scanning utilizing Multidetector Computed Tomography (MDCT) can detect early atherosclerosis in coronary arteries by identifying the amount of calcifications. In our study we employed these tools to detect occult atherosclerosis at least 5 years following breast RT.

**Methods:** We evaluated 20 asymptomatic patients, <60 years old, treated with RT at least 5 years prior to enrollment. Nine received RT to the left and 11 to the right chest wall. The median interval between RT and calcium scan was 8 years. All patients were treated with external beam RT using tangential technique. All patients underwent MDCT to compute volumetric and Agatston calcium scores of the coronary arteries and the aorta.

**Results:** Eleven patients had RT to the right chest wall, and eight had a calcium score of 0, while two had minimally elevated scores and one patient had a significantly elevated score. Meanwhile nine patients had RT to the left chest wall, and seven had a calcium score of 0. None had significantly elevated scores. In the aorta, 11 of 20 patients had a score of 0, while 8 of 20 had minimally elevated scores.

**Conclusion:** In contrast to studies demonstrating increased cardiovascular morbidity, our pilot study did not detect significant occult atherosclerosis using MDCT of the coronaries and aorta of patients assessed five or more years following radiation for treatment of breast cancer.

## Introduction

Radiation therapy (RT) is a crucial part of standard adjuvant therapy for breast cancer. Following publication of the NSABP B06 study in 1985, which demonstrated equivalence of breast conservation surgery plus radiotherapy to modified radical mastectomy, many women with early stage breast cancer have opted for breast conservation. There are more than 2.5 million women in the United States with a history of radiation exposure and are at long term risk for cardiovascular disease (1). Several studies have shown that these patients have an increased risk of morbidity and mortality from ischemic heart disease (2, 3). Most of these studies were done on patients treated between 1960 and 1980. More modern radiation techniques such as tangential fields reduce the heart and aorta’s exposure to ionizing radiation, and subsequent studies suggest a reduction in the risk of cardiovascular disease, however longer follow up is lacking (4, 5).

Aforementioned studies examined the late and irreversible effects of radiation on the cardiovascular system. One major mechanism of radiation induced cardiac damage is accelerated atherosclerosis and there are several screening tests available to detect early atherosclerosis. Electron Beam CT and now Multidetector CT provides a rapid, non-invasive method for identifying coronary artery calcifications from early atherosclerosis (6–8). The Agatston calcium score (AGS) and the Calcium Volume Score (CVS) are two methods of calcium score standardization that define levels of calcium burden. Both scoring systems are prognostic of future coronary cardiac events, and allow for the detection of asymptomatic patients at risk for coronary disease.

To our knowledge, coronary CT calcium scores have never been used to estimate coronary disease in asymptomatic patients previously treated with RT for breast cancer. We designed a study to evaluate the coronary calcium score in this population and correlate these findings with the laterality of cancer and with the patient’s family history and risk factors. Because atherosclerosis is a late effect of radiation, all patients included in our study had a history of radiation at least 5 years prior to CT assessment. Due to greater inclusion of the heart in the radiation portals of left-sided breast cancers compared to the right, coronary CT presented a unique opportunity to study differential atherosclerosis due to laterality of breast cancer after RT.

## Materials and Methods

### Study design

The study population comprised of 20 patients who were treated with RT for breast cancer. In an effort to exclude the confounding issue of increased atherosclerosis that occurs naturally with age, we only included patients who were 60 years of age or younger at the time of radiation. Only those patients who had radiation at least 5 years before enrollment were eligible. Patients were ineligible if clinical symptoms of coronary artery disease were present on initial history. Upon enrollment, each patient had a complete clinical history taken, a laboratory work-up including lipid panel, and a CT of the heart to determine the calcium scores of the coronary arteries and aorta by both AGS and CVS. The method of score calculation has been previously reported in detail (9). A cardiologist with expertise in cardiac imaging, who was blinded to the patient’s laterality of breast cancer, evaluated each CT. This study was approved by the Institutional Review Board and informed consent was obtained on every patient.

### Patient characteristics

Twenty patients who were previously treated for breast cancer with radiotherapy were included in the study (Table 1). Median age was 50 years at the time of enrollment (range 39–60). All patients were treated with external beam radiation using tangential techniques. Treatment dosing ranged 4500–5040 cGy using 6 MV photons. The fraction size was 200 cGy/day in patients whose separation from medial to lateral beam entry point was 24 cm or less, and was 180 cGy/day for separations >24 cm. An independent jaw technique was used to reduce divergence into the underlying lung and heart. A separate heart block was used if needed at the inferior tangential border of left-sided treatments to keep the amount of left ventricle irradiation to <10% of the total planned dose. The tumor bed was boosted to a total dose of 6000–6600 cGy depending on final margin status using 6–12 MeV electrons. Wedges were used during treatment to keep the dose within 5% of the prescribed dose. Patients had CT simulation for radiation planning. The heart was contoured and constraints per RTOG protocol guidelines were followed to limit cardiac dose. Dosimetrists were instructed to keep ≤5% of the heart to ≥20 Gy for left-sided tumors and 0% of the heart should receive ≥20 Gy for right-sided tumors.

**Table 1:** **Patient characteristics**.

Patient no.	Age (years)	Age at RT	Interval (years)^a^	Left vs. right breast cancer	RT dose (cGy)	RT boost to tumor bed (cGy)	Risk factors for coronary artery disease
1	51	45	6	LEFT	4975	1000	Lipids
2	43	35	8	LEFT	4500	1600	Lipids, HTN, FHx^b^
3	54	48	6	LEFT	4600	1800	Lipids
4	52	47	5	LEFT	4680	1400	Lipids, tobacco
5	43	36	7	LEFT	4600	2000	Lipids, FHx
6	54	49	5	LEFT	5040	1000	Lipids, FHx
7	39	34	5	LEFT	5000	1600^c^	Lipids, tobacco
8	56	50	6	LEFT	4600	1000	Lipids
9	51	43	8	LEFT	5000	0^c^	None
10	60	52	8	RIGHT	4600	1400	Lipids, HTN, FHx
11	51	43	8	RIGHT	5000	0^d^	Lipids
12	50	45	5	RIGHT	5040	0^c^	None
13	54	48	6	RIGHT	4680	1400	None
14	53	43	10	RIGHT	4600	1400	Lipids, HTN
15	40	30	10	RIGHT	4600	1400	None
16	48	40	8	RIGHT	4500	1600	Lipids
17	47	35	12	RIGHT	4600	1000	Lipids
18	55	47	8	RIGHT	5040	0^e^	Lipids
19	52	38	14	RIGHT	4600	0	Lipids
20	57	48	9	RIGHT	4600	0	None

^a^Time period between RT and coronary CT.

^b^FHx, family history of CAD.

^c^4600 cGy given to left supraclavicular and left posterior axilla.

^d^4600 cGy given to internal mammary nodes (IMN).

^e^Radiation given to IMN and right supraclavicular fossa.

Nine patients (patients 1–9) were treated for left breast cancer and received radiation to the left breast and chest wall. The remaining 11 patients were treated for right breast cancer and received radiation to the right breast and chest wall (patients 10–20). Seven patients received radiation at an outside institution (1, 4, 6, 8, 16, 18, 20). Two patients received radiation to the internal mammary nodes (IMN). The median interval between the radiation and the cardiac CT screen was 8 years with a range of 5–14 years (Table 1).

No patients had symptoms of cardiac ischemia. Three patients had hypertension controlled with diet and medications. Fifteen patients had a history of hypercholesterolemia, in most cases treated with diet and exercise. Four patients had a strong family history of coronary artery disease. None of the patients had diabetes.

### Image acquisition parameters

A coronary CT with calcium score was performed in all patients. All studies were performed on a 64-multidetector computed tomography (MDCT) scanner (GE Healthcare). An unenhanced sequence was performed to assess the volumetric and Agatston coronary calcium scores [SmartScore (GE Healthcare); 120 kV; 440–500 mA; rotation time, 0.35 s]. The CT scans were evaluated by a cardiologist who was blinded to the patient’s breast cancer and radiation history.

## Results

Of the nine patients who received radiation to the left breast and chest wall, none had significantly elevated calcium scores. Two patients were found with minimal calcium scores (Patients 1 and 7). Both had hyperlipidemia and one had a history of smoking. The remaining seven patients had a calcium score of 0 (Table 2).

**Table 2:** **Electron beam CT calcium scores**.

Patient no.	Age (years)	Left main coronary artery		Left anterior descending artery (LAD)		Right coronary artery (RCA)		Left circumflex artery (LCX)		Ascending aorta		Descending aorta	
		AGA^a^	CVS^b^	AGA	CVS	AGA	CVS	AGA	CVS	AGA	CVS	AGA	CVS
1	51	0	0	3	6	0	0	0	0	0	0	3	9
2	43	0	0	0	0	0	0	0	0	0	0	0	0
3	54	0	0	0	0	0	0	0	0	0	0	0	0
4	52	0	0	0	0	0	0	0	0	0	0	0	0
5	43	0	0	0	0	0	0	0	0	0	0	0	0
6	54	0	0	0	0	0	0	0	0	2	7	0	0
7	39	0	0	0	0	1	4	0	0	3	10	0	0
8	56	0	0	0	0	0	0	0	0	6	19	0	0
9	51	0	0	0	0	0	0	0	0	0	0	0	0
10	60	0	0	0	0	7	11	0	0	4	11	0	0
11	51	0	0	0	0	0	0	0	0	0	0	0	0
12	50	0	0	0	0	0	0	0	0	0	0	0	0
13	54	0	0	0	0	0	0	0	0	4	13	0	0
14	53	0	0	0	0	0	0	0	0	0	0	0	0
15	40	0	0	0	0	0	0	0	0	0	0	0	0
16	48	0	0	0	0	0	0	0	0	0	0	0	0
17	47	0	0	0	0	0	0	0	0	0	0	8	23
18	55	0	0	1	4	0	0	0	0	0	0	0	0
19	52	0	0	176	225	31	30	127	101	0	0	18	18
20	52	0	0	0	0	0	0	0	0	0	0	0	0

^a^AGA, Agatston score.

^b^CVS, calcium volume score.

Of the 11 patients who had radiation to the right breast and chest wall, 8 had coronary calcium scores of 0. Two patients had minimal calcium scores (patients 10 and 18), both had hyperlipidemia and one had hypertension and a strong family history of CAD. Only one patient had a significantly elevated calcium score (patient 19) that was above the 90th percentile for her age and sex for her AGS and CVS in the LAD and LCX. Surprisingly, she had a right breast cancer and received adjuvant radiation to the right breast and chest wall. She was only 55 years old and had no history or smoking or diabetes. Her interval between radiation and the screening CT was 14 years. She had hypercholesterolemia with a cholesterol of 236 and LDL of 154 and a history of inflammatory bowel disease. She was subsequently referred to have a cardiac work up.

Calcium scores in the descending and ascending aorta were also evaluated. Of the 20, only 8 patients had an elevated calcium score, but none were above 25.

## Discussion

### Significant findings

The coronary calcium score is a screening tool intended to detect asymptomatic coronary atherosclerosis, and its prognostic value for future coronary events has been validated (10). Years of accumulated data point to excessive cardiac mortality in women who have received adjuvant radiation for breast cancer or ductal carcinoma *in situ* (DCIS), and it is likely that radiation induced accelerated coronary artery and aortic atherosclerotic disease is contributory to this. Indeed, the calcium score has been recently shown by Rademaker et al. (11) to be a useful tool in the evaluation of radiation-related CAD in asymptomatic patients treated with mediastinal irradiation for Hodgkin’s Lymphoma. This study showed eight of the nine patients to have coronary atherosclerosis with high Agatston scores.

We therefore designed a pilot study to assess the delayed effects of adjuvant radiation for breast cancer. To our knowledge, this study is the first to report calcium scores to estimate coronary and aortic disease in patients previously treated with RT for breast cancer. Special attention was made to note the laterality of radiation exposure, personal and family history of risk factors, and time interval between radiation and calcium score screening.

The AGS and the CVS were both used for calcium scoring on each patient. Current guidelines for interpretation of coronary artery calcium scores (12) suggest categorizing risk groups by scores from 0 (very low risk), 1–10 (low risk), 11–100 (moderate risk), 101–400 (moderately high risk), to >400 (high risk). After a median of 8 years post adjuvant RT for DCIS or early breast cancer, only 1 among 20 women had a calcium score higher than 25 for any vessel (CVS 225 to the LAD). She was 52 years old at the time of study, and was 38 when she was diagnosed with right-sided early breast cancer and had lumpectomy followed by 4600 cGy given to the right chest wall with no boost to the tumor bed. Her medical history was only pertinent for hyperlipidemia, and had no other personal or family history of other risk factors.

This patient had the longest interval between radiation exposure and time of coronary calcium scanning (14 years). This finding may suggest that a longer minimum screening interval may be necessary to detect radiation induced atherosclerotic disease. However, the patient with the next longest interval was 12 years (patient 17), and her case is remarkably similar in that she was diagnosed at age 35, and had calcium screening at age 47. She also had a right-sided breast tumor and was treated with 4600 cGy followed by an additional 1000 cGy boost to the tumor bed. She too had only a history of hyperlipidemia, without personal or family history of other risk factors. Despite similar age at presentation, similar interval of radiation to screening time, and having had 1000 cGy more radiation, her highest calcium score was 25 (CVS) to the descending aorta.

Meanwhile, 11 of the 20 patients had a calcium score of 0 for all vessels. These included five patients with up to 5000 cGy to the left side, and up to 2000 cGy additional boost to the left-sided tumor bed. Additionally, one of these 11 patients had a smoking history and two others had a significant family history of cardiac disease. Most of the remaining patients had no calcium scores more than 10, reflecting minimal calcium burden and risk.

Given the proximity of the heart and great vessels to the left-sided radiation portals – even accounting for tangential field techniques – it is still notable that none of the patients with left-sided breast cancer or DCIS had a significantly high calcium score. None had a coronary calcium score >10 (Range 0–4), and none had an aortic calcium score >20 (Range 0–19), among the patients who had left-sided breast disease.

### Historical correlation

Historically, most cardiac disease has been observed in patients receiving radiation to the left chest wall (3, 4, 12, 13). Cause-specific mortality by breast cancer laterality was analyzed in 54,617 patients reported to the Swedish Cancer Registry during 1970–1985. After a median follow up of 9 years, patients with left-sided tumors were found to have a higher mortality due to MI, although, total cardiovascular and inter-current mortality was not different in both groups (3). In an epidemiologic cohort study, 300,000 US women were followed prospectively for cause-specific mortality and a higher risk of cardiac death was observed among patients with left-sided breast cancer than those with right-sided breast cancer, and this risk increased with time since treatment (14).

In a recent population-based case-control study involving more than 2100 women who received adjuvant radiation for breast cancer between 1958 and 2001, there was a significant increase in major coronary events for the left side vs. right-sided radiation exposure (15). A linear relationship between radiation exposure and rate of coronary events was found: 7.4% per gray (95% CI 2.9–14.5; *p* < 0.001), and the effects began at 5 years post-radiation, and continued for at least 20 years.

Therefore, recognition of the adverse effects of older radiotherapy techniques has led to increased efforts to avoid inclusion of the heart in the radiation field and the elimination of the gamma radiation fields that were used to treat IMN. The change in delivery of radiation via linear accelerators alone has been shown to substantially decrease radiation to the heart (16). Additionally, the use of tangential fields was shown to avoid direct heart exposure in the radiation field in more than 95% of patients (17). Many of the above studies include women treated with older technique.

All patients included in our study were treated after 1990, with modern techniques. Only 2 out of 20 received IMN radiation (Patients #11 and #18), and neither patient had a calcium score >10 of any vessel. The overall lack of adverse coronary artery findings may suggest that concerns about radiation induced CAD in breast cancer may not be applicable to current radiation techniques.

It is unclear why only one patient had a significant calcium score, and that contrary to expectations, she had no significant personal or family history of risk factors, except for hyperlipidemia. It is possible there are yet unknown factors that lead to accelerated atherosclerosis outside of traditional risk factors. Indeed, there is growing evidence that the coronary calcium score serves as an independent prognostic risk factor for coronary events and mortality, irrespective of other traditional risk factors (18). In this cohort study of 44,052 patients, individuals without any traditional risk factors but with an elevated calcium score had a significantly higher all-cause mortality rate than those who had three or more risk factors, but had coronary calcium scores of 0. Thus there may not be a direct correlation between having more risk factors and having a higher coronary calcium score.

### Limitations

Our study is limited by both size and duration of post-radiation followup. No conclusion can be drawn from a small sample size, however our findings may yield cautious optimism that modern radiation techniques that minimize exposure to the heart and great vessels may indeed reduce major cardiac events. The one patient with a moderately elevated calcium score had radiation to the right chest wall, and it is extremely unlikely that her calcium score is related to her adjuvant radiation.

Despite a median of 8 years post-radiation followup time in our study, we take note that the Swedish Cancer Registry study (3) was able to demonstrate cause-specific mortality with a median 9 year followup, in women who had radiation for left-sided vs. right-sided breast cancers. Furthermore, the recent population-based case-control data published by Darby et al. suggests that major cardiac events occur as early as 5 years post-radiation exposure. Thus we may reasonably anticipate elevations in coronary and aortic calcium scores within the minimum 5 years followup in our present study.

Other limitations are focused on the calcium scoring mechanism and the screening technology. Non-calcified plaques are not detected by EBCT and this component of atherosclerosis may be overlooked. Also, newer, alternative calcium scoring methods are being devised which may be superior to the AGS and CVS. The Calcium Mass score has been touted as more accurate than the AGS and CVS (19, 20). Also, lesion and vessel specific coronary artery calcium scoring has been shown to be superior to Agatston in the detection of obstructive coronary artery disease (21). Despite the superior theoretical improvements in calcium detection with the newer calculation methods, a study that directly compared the Agatston, Calcium Volume, and Mass scores of 11,490 patients found no significant difference in agreement between the three methods (20).

## Conclusion

In conclusion, this pilot study of 20 women did not find an increase in coronary or aortic calcium burden by AGS or CVS in patients treated with left-sided vs. right-sided chest wall radiation for adjuvant therapy for early breast cancer or DCIS. This is contrary to earlier data, and it is possible that newer radiation techniques that minimize radiation exposure to the heart may account for our findings. Larger, prospective trials with longer followup are needed to clarify the role of coronary CT calcium scores in evaluating and monitoring patients with prior breast irradiation.

## Conflict of Interest Statement

The authors declare that the research was conducted in the absence of any commercial or financial relationships that could be construed as a potential conflict of interest.
